# Pain assessment in intensive care: who puts pen to paper?

**DOI:** 10.1186/2197-425X-3-S1-A328

**Published:** 2015-10-01

**Authors:** H Laycock, H Wordsworth, Pan-London Peri-operative Research and Audit Network (PLAN) and South East Anaesthetic Research CHain (SEARCH)

**Affiliations:** Imperial College, London, United Kingdom

## Introduction

Pain is common in intensive care units (ICUs)[1]. Regular pain assessment can improve patient satisfaction and clinical outcomes. It is commonly performed by nurses [2], yet physician-led assessment can improve analgesic management. Using pain documentation as a surrogate for assessment, a review showed physiological parameters i.e. cardiovascular assessment, were more frequently documented by doctors than pain in critically ill patients [3].

## Objectives

Review pain documentation in ICU.

## Methods

An observational audit of adult ICU patients was conducted by trainee research networks (London and South East England). Governance approval was obtained at each hospital. Data collection was performed over two 24-hour periods (each representing a patient “episode”), with patients contributing data to either one or both periods. Medical and nursing notes were reviewed for pain and physiological parameter assessment and demographic data.

## Results

44 ICU's participated (including medical, surgical and specialist units). 1022 patient episodes were reviewed, contributing 2463 separate patient assessments conducted by 412 doctors. 712 separate patients were included (mean age 61.8 years, range 19-103 years). Patients were intubated in 38% of patient episodes (unable to self report).

### Nursing Assessments

29% of patient episodes had no nursing pain assessment. Pain assessment for both sedated and awake patients, commonly used a 0-3 scale (52%) or the numerical rating scale (19%). 0.02% of nursing pain assessments used validated assessment tools for sedated patients i.e. the Behavioural Pain Score or Critical-Care Pain Observation Tool.

### Doctor Assessment

79% of patient assessments documented by doctors included a cardiovascular system (CVS) review compared with 21% for pain. Of those pain assessments made, 89% described pain in terms of comfort or stability whilst 9% used a pain assessment tool.

## Conclusions

This large data set represents practice occurring in ICUs in South East England and London. Whilst pain poses significant physiological and psychological consequences for the critically ill, our results suggest a sizeable proportion lacked any pain assessment. Nursing assessment often used non-validated tools for patients unable to self-report. Doctors' documented CVS reviews 3 times more often than pain. Pain, when reported was mostly narrative. Authors recognise limitations include that a lack of documentation does not exclude an assessment, but believe it is a useful surrogate when evaluating patient care from charts. Our data raises a number of themes including whether doctors prioritise pain and the use of appropriate pain assessment tools in ICU. Does omission of pain documentation represent an assumption that pain management lies outside the remit of the doctor role, or perhaps reflects a lack of education?

## Acknowledgement

Dr F Rubulotta and Dr C Bantel

## References

[1] Payen : 2007

[2] Gelinas : 2004

[3] Laycock : 2015

